# RNA Sequencing of Contaminated Seeds Reveals the State of the Seed Permissive for Pre-Harvest Aflatoxin Contamination and Points to a Potential Susceptibility Factor

**DOI:** 10.3390/toxins8110317

**Published:** 2016-11-03

**Authors:** Josh Clevenger, Kathleen Marasigan, Vasileios Liakos, Victor Sobolev, George Vellidis, Corley Holbrook, Peggy Ozias-Akins

**Affiliations:** 1 Department of Horticulture and Institute of Plant Breeding, Genetics & Genomics, The University of Georgia, Tifton, GA 31793, USA; jclev@uga.edu (J.C.); kath1290@uga.edu (K.M.); 2 Department of Crop and Soil Sciences, The University of Georgia, Tifton, GA 31793, USA; vliakos@uga.edu (V.L.); yiorgos@uga.edu (G.V.); 3 USDA-ARS National Peanut Research Laboratory, Dawson, GA 39842, USA; victor.sobolev@ars.usda.gov; 4 USDA-ARS, Crop Genetics and Breeding Res. Unit, Tifton, GA 31793, USA; corley.holbrook@ars.usda.gov

**Keywords:** *Arachis hypogaea*, *Aspergillus*, aflatoxin, RNA-seq, non-coding RNA, uHPLC

## Abstract

Pre-harvest aflatoxin contamination (PAC) is a major problem facing peanut production worldwide. Produced by the ubiquitous soil fungus, *Aspergillus flavus*, aflatoxin is the most naturally occurring known carcinogen. The interaction between fungus and host resulting in PAC is complex, and breeding for PAC resistance has been slow. It has been shown that aflatoxin production can be induced by applying drought stress as peanut seeds mature. We have implemented an automated rainout shelter that controls temperature and moisture in the root and peg zone to induce aflatoxin production. Using polymerase chain reaction (PCR) and high performance liquid chromatography (HPLC), seeds meeting the following conditions were selected: infected with *Aspergillus flavus* and contaminated with aflatoxin; and not contaminated with aflatoxin. RNA sequencing analysis revealed groups of genes that describe the transcriptional state of contaminated vs. uncontaminated seed. These data suggest that fatty acid biosynthesis and abscisic acid (ABA) signaling are altered in contaminated seeds and point to a potential susceptibility factor, *ABR1*, as a repressor of ABA signaling that may play a role in permitting PAC.

## 1. Introduction

*Aspergillus flavus* and *Aspergillus parasiticus*, ubiquitous filamentous soil fungi, are a major threat to food security. During the interaction with many oil seed crops, *Aspergillus* spp. can produce aflatoxin, perhaps the most carcinogenic, naturally occurring compound known [1]. Aflatoxin contamination can occur during pre-harvest or post-harvest. Due to poor storage conditions, post-harvest contamination has contributed to outbreaks that have killed hundreds and affected thousands in developing countries [2,3,4]. Chronic exposure to low levels of aflatoxin has been linked to child stunting and hepatosplenomegaly, and enlargement of the liver and spleen [4,5]. Pre-harvest contamination is more subtle and can cause major yield losses for growers in developed countries. Limits of aflatoxin for human consumption are 20 ppb, and are even lower in Europe at 4 ppb. One sample over the limit in an entire lot can result in rejection. Estimates for the cost of aflatoxin in just southeastern US were $25 million per year from 1993 to 1996 [6].

Resistance to pre-harvest aflatoxin contamination (PAC) can result from different mechanisms. These mechanisms can be grouped into two different categories, resistance to *Aspergillus* invasion of the seed and resistance to aflatoxin production. Resistance to invasion of the seed can be deployed either mechanically, by seed coat structure or resistance to pest-induced pod damage, or molecularly, by increased defense response. Resistance to aflatoxin production can occur from differential expression of specific lipoxygenases or differences in fatty acid content of the seed [7,8]. A peanut 9*S*-lipoxygenase was found to be responsive to *Aspergillus* infection, whereas two 13*S*-lipoxygenases were shown to be repressed by infection [9,10]. This observation is intuitive with the observation that the products of 13*S*-lipoxygenases inhibit mycotoxin production while the products of 9S-lipoxygenases stimulate mycotoxin production [8,10,11], although recent evidence suggests this interaction is dependent on concentration [12]. In maize, it has been established that plant lipoxygenases play a role in the fungal-seed interaction that produced aflatoxin, as mutant plants with disrupted ZmLox3 were significantly more susceptible to aflatoxin production in vitro and in the field [13].

The fatty acid composition of the peanut seed has been implicated in differences in aflatoxin production [8]. Using isolines that differ by a mutation causing high oleic acid content, it was determined in vitro that the high oleic lines had significantly higher aflatoxin contamination when inoculated with A. flavus [8]. In the field, however, lines with low levels of linoleic acid showed no significant difference in aflatoxin contamination after being subjected to drought stress over three years and two locations [14]. These two studies, however do not contradict each other, because the complexity of the pre-harvest fungal-seed interaction is determined by many factors on a field plot level.

Drought tolerance has been studied as a correlative trait for aflatoxin resistance [15,16,17,18,19,20,21]. Holbrook et al., 2000 [18] proposed that drought tolerance-related traits could be used as an indirect method of selection for aflatoxin resistance after observing significant correlations between aflatoxin contamination and leaf temperature as well as visual stress rating. Arunyanark et al., 2009 [19] showed significant correlations between aflatoxin contamination and specific leaf area under drought stress, and showed that drought tolerant genotypes under severe drought had significantly less aflatoxin contamination. Despite this, the pod yield ratio between irrigated conditions and drought stress does not appear to be correlated with aflatoxin contamination [22].

Several RNA sequencing (RNA-seq) studies have been carried out to determine the genetic regulation of the peanut-*Aspergillus* pre-harvest interaction that leads to aflatoxin production in the field. Guo et al., 2008 [23] profiled expressed sequence tags from developing seeds of two genotypes in the field subjected to drought stress and *A. parasiticus* challenge. They used a putative ‘resistant’ genotype, GTC-20, and a susceptible genotype, Tifrunner. They found that certain defense-related and drought-responsive genes were up-regulated under *Aspergillus* challenge. Guo et al., 2011 [24] carried out a similar experiment, but used microarrays to profile expression. Neither study determined the aflatoxin content of the seeds used for expression analysis. Due to the complexity of the interaction, it is important to know if a seed is contaminated with aflatoxin in order to properly examine any differences in expression. Pooling of many seeds necessarily combines contaminated seeds with uncontaminated seeds with or without *Aspergillus* infection. The mosaic of seeds in a field plot necessitates a higher resolution approach to the selection of samples for sequencing. 

Despite the paucity of RNA-seq experiments in peanuts, studies have been done in cotton and maize. In cotton, inoculated bolls were harvested in a time series after inoculation and subjected to RNA sequencing [25]. Differential expression analysis found an up-regulation of defense-related and antifungal genes in pericarp and seeds in response to *Aspergillus* [25]. Dolezal et al., 2014 [26] inoculated maize kernels and profiled expression with a maize microarray at four days after infection. They observed an up-regulation of defense-responsive genes as well, but also observed a reprogramming of carbohydrate utilization [26]. Tang and colleagues [27] used association analysis to find enriched pathways for aflatoxin resistance in maize. They found a significant enrichment associated with single nucleotide polymorphisms (SNPs) within genes participating in the jasmonic acid (JA) biosynthesis pathway, including lipoxygenases. Additionally, they found non-pathway related genes associated with large effects on aflatoxin contamination, including defense-related genes such as leucine-rich repeat protein kinase (LRRPK), expansin B3 (EXPB3), and reversion-to-ethylene sensitivity1 (RTE1) [27].

To understand the genetic state of the peanut seed that is permissive or repressive of aflatoxin production, a highly controlled automated rainout shelter was used to select samples for RNA sequencing to profile expression during the interaction of the peanut seed and *Aspergillus*. Seeds were selected at yellow-2 maturity stage from six different genotypes and each seed was tested for the presence of *Aspergillus* and aflatoxin contamination. RNA-seq then was conducted to determine the genetic state that permits or represses aflatoxin production irrespective of genotype. 

## 2. Results

### 2.1. Prediction of Aflatoxin by Drought Tolerance-Related Traits

**Figure 1:**
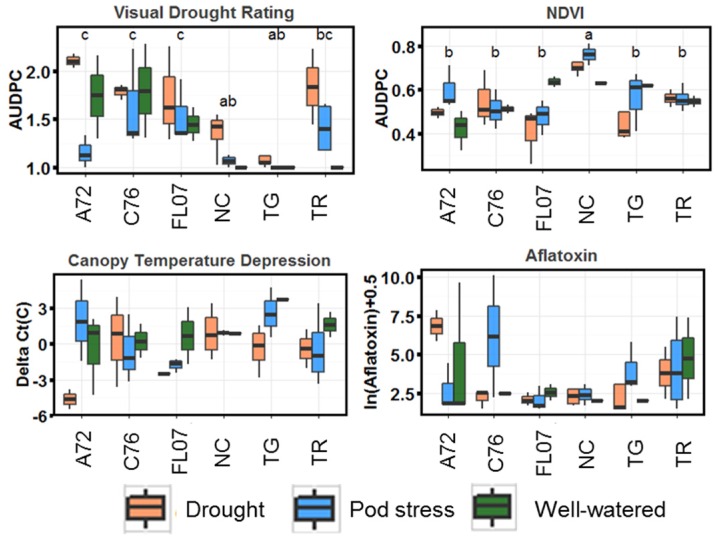
Box plots showing daily area under the drought progress curve (AUDPC) of drought tolerance-related traits during the 40-day stress period. Letters indicate significant differences by a Kruskal–Wallis test followed by Dunn’s test for comparisons of mean rank sums. Aflatoxin values were measured one time after harvest and were natural log transformed to stabilize variance. C76 = C76-16; FL07 = Florida07; NC = NC 3033; TG = Tifguard; TR = Tifrunner.

During the 40-day induced drought period, drought tolerance-related data were collected on all plots (Figure 1), including canopy Normalized Difference Vegetation Index (NDVI), canopy temperature depression (∆Ct), and visual rating data. 

Only visual rating and NDVI showed differences among genotypes using a non-parametric Kruskal–Wallis test, with Tifguard and NC 3033 performing the best for visual rating and NC 3033 having the highest NDVI over the drought treatment period. There was no difference by genotype for ∆Ct, although certain genotypes were significantly affected by drought treatment, including Florida-07 and A72.

Principal components analysis showed that 60% of the variation can be explained by the root and pod zone moisture (PC2; 22%) and ∆Ct, NDVI, and visual rating measurements (PC1; 38%) (Figure S1). Although there is no significant separation by treatment, there is a trend that is explained by PC2 (pod and root moisture). Aflatoxin level is partially explained by the combination of moisture level and ∆Ct and NDVI measurements.

Using linear modelling, we attempted to find the best model to predict aflatoxin contamination using the environmental data (Table S1). Treatment alone could not predict aflatoxin production (*R*^2^ = 0.0, *p* = 0.99). Canopy temperature depression (∆Ct) and genotype were the best predictors, although only ∆Ct had a significant model (*R*^2^ = 0.14, *p* = 0.01). The best model to predict aflatoxin production was~∆Ct + genotype + root zone moisture (*R*^2^ = 0.21, *p* = 0.02). Although not a strong correlation, the predictive *R*^2^ is higher (0.32) (Figure S2). Although these data do not show that the genotypes assayed can be described as resistant or susceptible to aflatoxin, the fact that each plot can maintain canopy temperature under drought stress and the soil moisture during the seed–*Aspergillus* interaction can predict a proportion of the variance due to aflatoxin, is encouraging. 

### 2.2. Differentially Expressed Genes in Seeds Contaminated with Aflatoxin

**Figure 2:**
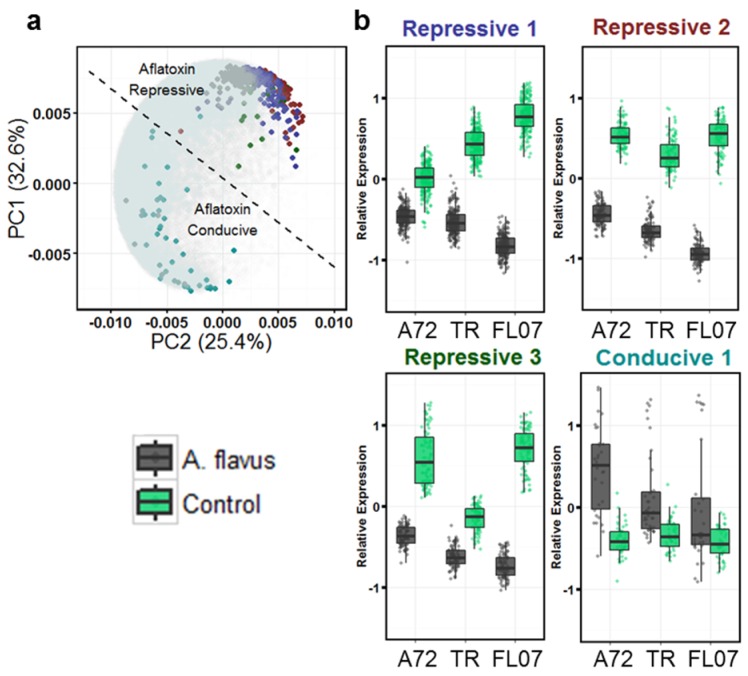
Differential expression of genes affected by *Aspergillus flavus* colonization and aflatoxin presence. (**a**): first two principal components of all gene expression profiles across all genotypes with the four identified groups highlighted with a different color. PC1 and PC2 (58% of variation) can define the two gene expression responses; (**b**): Four groups of differentially expressed genes. Scale is mean-centered relative expression. The color of the titles of the boxplots corresponds to highlighted points in A. TR = Tifrunner; FL07 = Florida-07. Expression is the mean centered normalized z-score of the average FPKM (Fragments per Kilobase of exon per Million reads Mapped) of 2–3 biological replicates each made up of two pooled seeds. Black indicates the presence of *Aspergillus* spp. and aflatoxin on the seeds. Green indicates no *Aspergillus* spp. present on the seeds.

The effect of aflatoxin contamination across all three genotypes with contaminated seeds was tested. Control seeds to test *A. flavus* invasion were seeds positive for *Aspergillus* but not contaminated with aflatoxin. Controls seeds to test aflatoxin production were seeds positive for *Aspergillus* and contaminated with aflatoxin. This test determined that 543 genes were differentially expressed due to aflatoxin contamination (Table 1) that could be clustered into four main groups (Figure 2a). 

**Figure 3:**
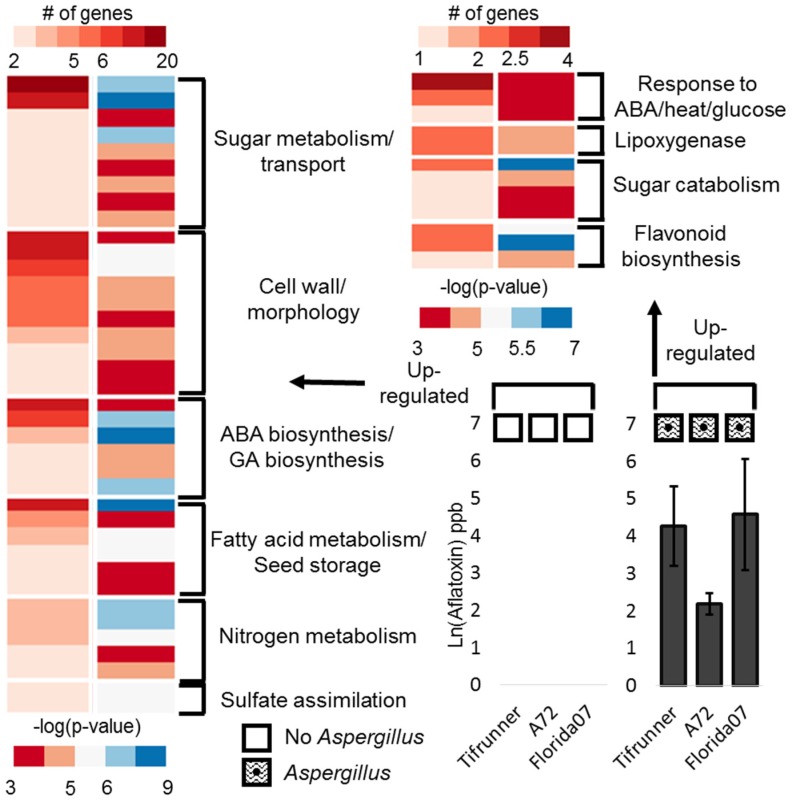
An enriched GO term analysis of differentially expressed genes. The left panel of each heatmap shows number of genes with an enriched GO term (top scale). The right panel of each heatmap shows the log transformed adjusted *p*-value from a hypergeometric enrichment test (bottom scale). GO terms are grouped into meta categories shown at the right of each heatmap. The left heatmap shows genes up-regulated in samples free of infection and contamination. The upper right heatmap shows genes up-regulated in samples contaminated with aflatoxin. Aflatoxin graphs show the natural log transformed average aflatoxin of pooled seeds with standard error. Zero values indicate no detection above detectable limits from those samples.

The three aflatoxin repressive groups contain genes that are up-regulated when a seed is free of *Aspergillus* invasion and aflatoxin contamination across all three genotypes compared with contaminated seeds. Enriched gene ontology (GO) term testing of all three groups revealed the signature of the expression in the seed that has escaped contamination (Figure 3). Organizing enriched GO terms by pathway showed that sugar metabolism/transport, cell wall restructuring and morphology, fatty acid metabolism, seed storage, ABA and gibberellic acid (GA) biosynthesis, nitrogen metabolism, and sulfate assimilation were up-regulated in seeds free of contamination. 

The aflatoxin conducive group represents a small set of genes that showed up-regulation in seeds contaminated with aflatoxin. There were a few pathways that showed enrichment, including genes responsive to ABA, glucose, and heat, flavonoid metabolism, sugar catabolism (fucosidase activity) and lipoxygenase activity (Figure 3). In addition, two genes related to a stress response, including Pathogenesis related 2, and *ABR1* were shown to be up-regulated in contaminated seeds.

**Figure 4:**
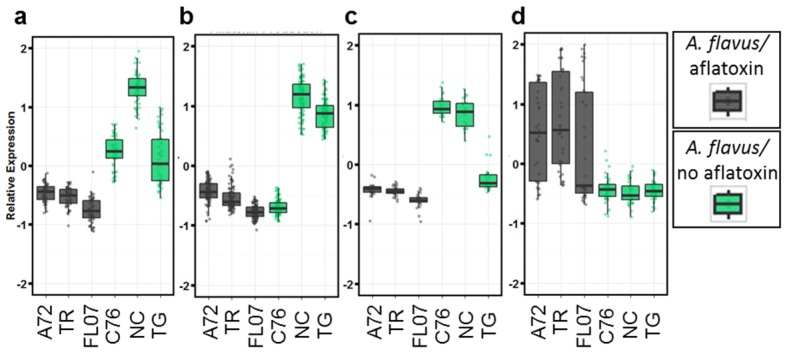
Groups of genes differentially expressed between aflatoxin contaminated seeds and seeds infected with *Aspergillus* but not contaminated with aflatoxin ignoring genotype, separated into four groups depending on expression profile across genotypes. (**a**) 53 genes up-regulated in all three genotypes with no contamination compared to contaminated seeds; (**b**) 182 genes up-regulated in only NC 3033 and Tifguard non-contaminated, infected seeds; (**c**) 24 genes up-regulated in only C76-16 and NC 3033 non-contaminated, infected seeds; (**d**) 29 up-regulated genes in all three genotypes with contaminated seeds compared to non-contaminated, infected seeds. C76 = C76-16; FL07 = Florida07; NC = NC 3033; TG = Tifguard; TR = Tifrunner. Expression is the mean centered normalized z-score of the average FPKM (Fragments per Kilobase of exon per Million reads Mapped) of 2–3 biological replicates each made up of two pooled seeds. Black indicates the presence of *Aspergillus* spp. and aflatoxin on the seeds. Green indicates *Aspergillus* spp. present but no aflatoxin contamination on the seeds.

The analysis of differentially expressed genes due to aflatoxin contamination when *Aspergillus* colonization is present yielded 506 genes (Table 1). These genes again were grouped according to expression pattern using self-organizing maps and yielded four distinct groups of interest (Figure 4). Three groups showed up-regulation in seeds colonized by *Aspergillus* that were not contaminated with aflatoxin, each with a different genotype showing the strongest difference (Figure 4a–c).

**Figure 5:**
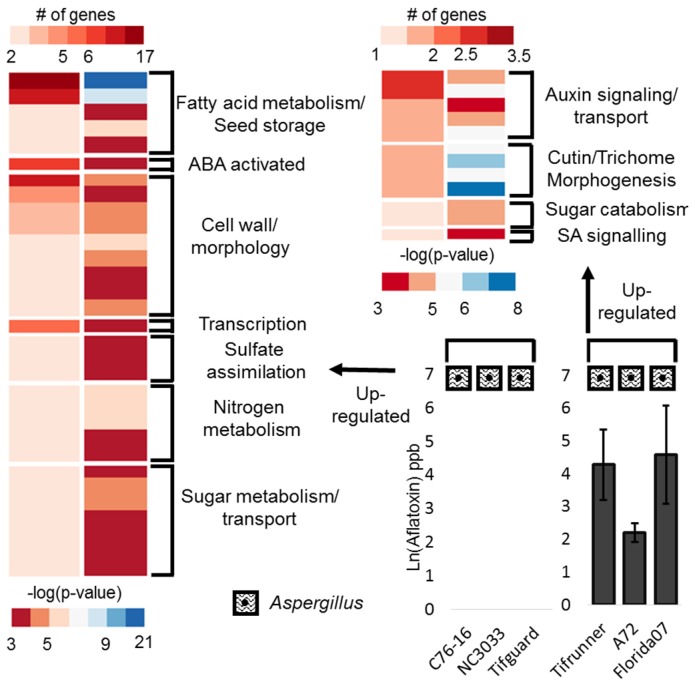
An enriched GO term analysis of differentially expressed genes. The left panel of each heatmap shows number of genes with an enriched GO term (top scale). The right panel of each heatmap shows the log transformed adjusted *p*-value from a hypergeometric enrichment test (bottom scale). GO terms are grouped into meta categories shown at the right of each heatmap. The left heatmap shows genes up-regulated in *Aspergillus* infected samples free of contamination. The upper heatmap shows genes up-regulated in samples contaminated with aflatoxin. Aflatoxin graphs show the natural log transformed average aflatoxin of pooled seeds with standard error. Zero values indicate no detection above detectable limits from those samples.

An enriched GO analysis of these three groups reveals a consistent picture with fatty acid metabolism, nutrient storage, cell wall restructuring and morphology, nitrogen metabolism, sugar metabolism and transport, ABA activation, and sulfate assimilation (Figure 5).

The group of genes up-regulated in the aflatoxin contaminated seeds/genotypes describe the enrichment of auxin signaling and transport, including a possible ortholog of the auxin transport gene, *BIG*, which is annotated with the GO term, “response to fungus”. Two heat shock proteins and an alpha-fucosidase are also up-regulated.

### 2.3. Aflatoxin Contamination Reveals Differential Expression of Non-Coding RNAs

**Figure 6:**
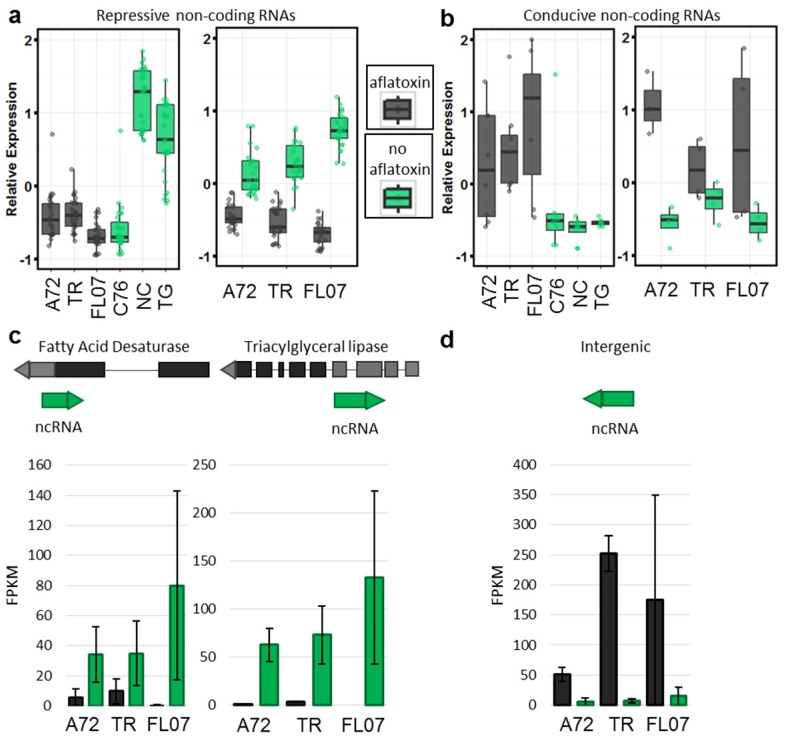
ncRNAs are differentially expressed in contaminated seeds. (**a**,**b**) groups of ncRNAs differentially expressed within genotypes (right) and between genotypes (left) differentiated by aflatoxin contamination; (**c**) Two examples of down-regulated antisense ncRNAs associated with fatty acid biosynthesis genes and their orientation within the predicted gene. Bar graphs below show average expression in FPKM with standard error of two replicates (contaminated) and three replicates (not contaminated); (**d**) One example of a ncRNA up-regulated in contaminated seeds is intergenic and not associated with a predicted gene. FL07 = Florida-07; TR = Tifrunner. Alignment of ncRNAs is relative to *A. duranensis* and *A. ipaensis* pseudomolecules and so is only an approximate alignment for *A. hypogaea*. Transcripts were assembled de novo and identified as putative non-coding independently.

We identified 35 differentially expressed ncRNAs with expression patterns of interest; 25 up-regulated when aflatoxin was not produced (repressive) and 10 up-regulated when aflatoxin was produced (conducive) (Figure 6). These ncRNAs were annotated based on their location in the genomes of *A. duranensis* and *A. ipaensis* relative to annotated genes (peanutbase.org, Ames, IA, USA) (Table S2). Of the 35, 21 were intergenic, and 14 were antisense exonic.

There were 25 ncRNAs up-regulated when aflatoxin was not produced (Figure 6A). Three of the antisense exonic ncRNAs were associated with annotated genes. Two were associated with stress-related genes, a proteinase inhibitor and a late embryogenesis abundant (LEA) family gene. The other was associated with a chloroplastic fatty acid desaturase, a putative *FAD2* homolog. Antisense transcription of ncRNAs can affect gene regulation of associated genes either transcriptionally or post-transcriptionally [28]. These ncRNAs may regulate gene expression in response to *Aspergillus* or developmentally.

There were 10 up-regulated ncRNAs when aflatoxin was produced (Figure 6B), although four were differentially expressed within the genotype and six between genotypes (Table S2). Unfortunately, the exonic ncRNAs in this group were associated with mostly unknown proteins. (Figure 6D).

### 2.4. SNPs Associated with Differentially Expressed Genes as Possible Aflatoxin-Associated eQTL

Using a specialized pipeline for identifying markers in polyploids called SWEEP [29], single nucleotide polymorphisms (SNPs) within transcripts (coding region) were identified between all genotypes used in this study, then associated with genes that showed unique differential expression. If a gene was differentially expressed in one genotype in response to *Aspergillus* invasion or aflatoxin contamination and that genotype had a unique SNP within that gene, that SNP was considered to be a possible aflatoxin-associated eQTL.

We identified 11 such possible eQTL; nine associated with Florida-07 and two associated with Tifrunner (Table 2). The fold change of the genotype with a significant change in expression between a contaminated seed and an uncontaminated seed was calculated, and then the average fold change of the other five genotypes was calculated. 

These potential expression quantitative trait loci (eQTL) are of interest because of the difference in genetic response. Figure S3 shows three genes of interest associated with a possible eQTL SNP: a sugar transporter, a receptor-like kinase *HSL1*, and an alpha-fucosidase. The sugar transporter is less expressed in seeds contaminated with aflatoxin, although significant only in Florida-07 (Table 2). Aflatoxin production is stimulated by the simple sugars sucrose and glucose, but not by more complex carbohydrates (Payne and Brown, 1998). The receptor-like kinase *HSL1* is also less expressed in aflatoxin contaminated seeds of Florida-07 and shows almost no change in expression in the five other genotypes (Table 2; Figure S3). The expression of the alpha-fucosidase is almost 3.5 times higher in aflatoxin contaminated Tifrunner seeds, but shows minimal change in the other five cultivars (Table 2; Figure S4).

## 3. Discussion

Pre-harvest aflatoxin contamination is extremely variable, making breeding for ‘resistance’ to contamination in the field difficult [30]. The sampling method can contribute to 90% of the variance between samples, and as sample size increases (in our case grams of seeds) standard deviation decreases [30]. In our experimental design, soil compaction and reliance on pods to develop in the isolated pod zone rather than near the tap root in the root zone necessarily decreased our sample size for plot level aflatoxin estimation. This observation leads to the conclusion that we cannot make a statement about the level of ‘resistance’ of the genotypes in our study. We must assume that each genotype will interact with *Aspergillus* spp. differently but the main differences of practical interest are between seeds showing aflatoxin contamination and those that remain uncontaminated. The drought treatments and drought-tolerance phenotyping can provide information about the ability of each genotype in this study to tolerate drought stress. Using the unbiased measurements of canopy temperature depression and NDVI, NC 3033 and C76-16 stand out as maintaining their health under drought stress when comparing between treatments. Perhaps as a coincidence, within both genotypes, no collected seed infected with *Aspergillus* was contaminated with aflatoxin above the detectable limit of 0.1 ppb.

For the first time, expression in peanut seeds in a field-like setting, that were contaminated with aflatoxin, was profiled. The transcriptional state of these contaminated seeds was compared with seeds that were not contaminated. The ‘control’ samples are not seeds from un-inoculated plots but are instead seeds that escaped contamination either with aflatoxin, *Aspergillus* infection, or both. RNA-seq experiments in the field are susceptible to high variability between replicates due to the myriad of environmental differences within each microenvironment. Although we were able to control soil temperature and moisture throughout the season, pest pressure and other abiotic factors could not be controlled beyond normal cultural practices. That being said, we were able to find genes differentially expressed due to *Aspergillus*/aflatoxin presence that maintained their trend across different genotypes. This small set of genes is the first step toward understanding the genetic state of a developing seed in the field that is repressive or permissive of aflatoxin contamination.

**Figure 7:**
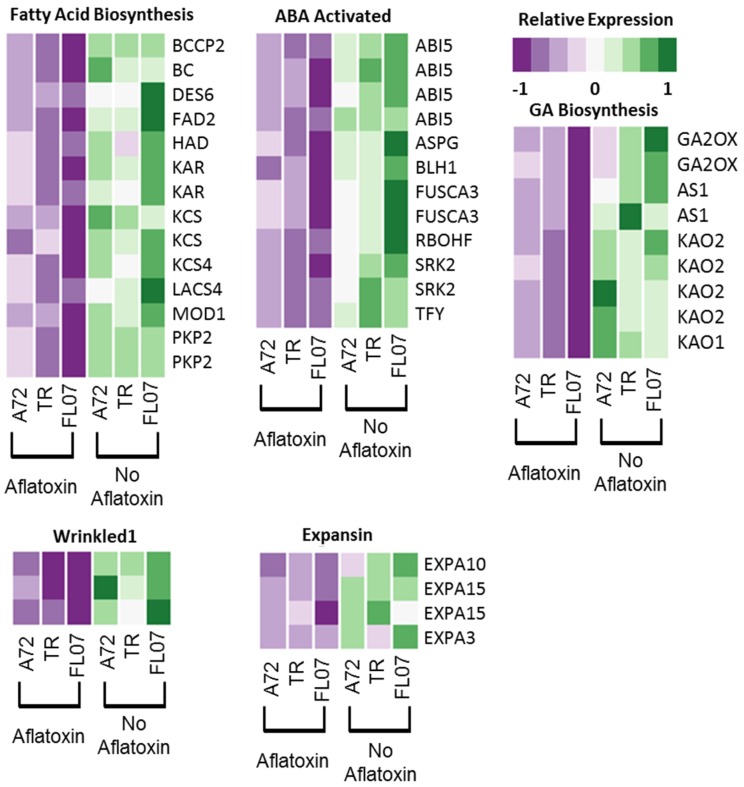
Pathways down-regulated in aflatoxin contaminated seeds. Heatmaps show the relative expression (*Z*-score normalized) of each gene shown to the right. Each heatmap is grouped into pathways.

In two different comparisons, certain pathways were revealed to be more highly expressed in seeds that escaped aflatoxin contamination. The most striking pathway was fatty acid biosynthesis with ten enzymes in the pathway down-regulated in contaminated seeds (Figure 7; Figure S4) including *FAD2*, *LACS*, *KAR*, *ENR*, *KAS1*, *BC*, *KCS*, *BCCP*, DES6, and *HAD*. Additionally, three orthologs of *WRINKLED1* were down-regulated in aflatoxin contaminated seeds (Figure 7). *WRINKLED1* is a transcription factor that controls fatty acid biosynthesis by binding to the promoters of enzymes in the fatty acid biosynthesis pathway to activate their expression [31,32].

*WRINKLED1* binds to enzymes found to be affected in this study, including *KAS*, *BCCP*, *ENR*, *DES*, *PKB*, and even sucrose synthase (*SUS*) [32]. For genotypes with *Aspergillus* infected seeds and no contamination, there was no expression difference seen compared to seeds with no *Aspergillus* infection (Figure S5). The data point to a possible difference in fatty acid content in aflatoxin contaminated seeds. In peanuts, the ratio of oleic acid to linoleic acid had a significant effect on post-harvest aflatoxin contamination [8]. In the field, plot level pre-harvest aflatoxin was not significantly different between cultivars with varying levels of linoleic acid [14] due to the high variance associated with pre-harvest aflatoxin contamination.

In this study, maturity was controlled by selecting only yellow-2 stage seeds. The difference in expression in the fatty acid biosynthesis pathway between aflatoxin contaminated seeds and seeds free of contamination is robust across three different genotypes. From this experiment, it is unclear whether the transcriptional state of the seed enabled aflatoxin production or was altered by the fungus. There is evidence that plant lipoxygenases can mimic fungal lipoxygenases in the seed–*Aspergillus* interaction and affect aflatoxin production [9,10,13]. Although the fatty acid biosynthesis pathway is altered in these data, there is no evidence that lipoxygenase expression is significantly altered. Four lipoxygenases were differentially expressed in this study, but two were up-regulated in aflatoxin contaminated seeds and two were down regulated. All four lipoxygenases were predicted to be 13S-lipoxygenases (data not shown) and so the data do not fit the hypothesis that 13S-lipoxygenases inhibit aflatoxin contamination in the field.

Down-regulation of the abscisic acid signaling pathway was also observed (Figure 7), including four copies of *ABSCISIC ACID INSENSITIVE5*, *ASPG1*, *BLH1*, both homeologs of *FUSCA3*, and *RESPIRATORY BURST OXIDASE HOMOLOG*. These genes play roles in ABA signaling and also sugar and stress response [33,34], seed maturation [35], fatty acid biosynthesis and seed oil production [36,37], drought avoidance [38], and ABA activated reactive oxygen species (ROS) production [39]. In the developing seed, ABA inhibits germination and promotes desiccation tolerance [33,40]. Whether this pathway is down-regulated in contaminated seeds because those seeds were not able to mature properly or alternatively because of reprogramming from *Aspergillus* infection is not clear. This pathway across three genotypes is down-regulated in seeds that are contaminated. Enhanced ABA signaling in developing seeds is a strong breeding candidate for increased seed tolerance to drought and avoidance of aflatoxin contamination.

Gibberellic acid (GA) and ABA play antagonistic roles during seed maturation [41]. In aflatoxin contaminated seeds, the GA pathway has been down-regulated (Figure 7). Gibberellic acid has been shown to promote seed coat development [42]. In these data, the mucilage metabolic process involved in seed coat development is also down-regulated in aflatoxin contaminated seeds, suggesting a possible seed coat deficiency in contaminated seeds compared to seeds that have escaped contamination.

Non-coding RNAs have an effect on regulation in diverse ways, pre- and post- transcriptionally. Mechanisms that have been shown experimentally include chromatin modification, transcriptional repression, inducing isoform variation, increasing translation efficiency, and increasing RNA stability [28]. In this study, we have identified certain ncRNAs that are down-regulated in aflatoxin contaminated seeds, and perhaps more interestingly, ncRNAs that are up-regulated in aflatoxin contaminated seeds. Some of these ncRNAs are intergenic, located in regions of the genome between genes. A few, however, reside in gene sequences, either overlapping exons and introns or overlapping exons and the 5′ or 3′ untranslated region (UTR). These genes associated with ncRNAs down-regulated in aflatoxin contaminated seeds include *fad2* and a lipase, two genes involved in fatty acid biosynthesis, as well as a proteinase inhibitor. Unfortunately, the ncRNAs up-regulated in aflatoxin contaminated seeds are intergenic and mostly associated with unknown genes. The analysis of RNA-seq data needs to include ncRNAs in order to capture a more complete regulatory picture. The ncRNAs identified in this study may play important roles in the *Aspergillus*–peanut interaction that is permissive for aflatoxin production and further research should be done to elucidate their function.

The small number of genes that are up-regulated in aflatoxin contaminated seeds are intriguing because they represent possible susceptibility factors. Unfortunately, there are few of these up-regulated genes and so these data are hard to interpret. Individually, the up-regulated genes include two 13S-lipoxygenases, both homeologs of ethylene-responsive transcription factor *ABR1*, two lectins, *PATHOGENESIS-RELATED 2*, a BAHD acyltransferase *DCR*, a deoxy-chalcone synthase, and two anthocyanidin-malonyltransferases. These genes point to a possible defense response. The sampled seeds were contaminated with aflatoxin, however, and so any possible defense response would be useless from the perspective of the farmer. Similar observations have been made from previous peanut studies [23,24]. Pre-harvest aflatoxin resistance in the field does not appear to be linked to an enhanced or specialized defense response in the seed. Elevated gene expression of defense-related genes is irrelevant in seeds that are already contaminated. 

The ethylene responsive transcription factor *ABR1* is very interesting because it is a repressor of ABA response [43]. In contaminated seeds, ABA signaling is down-regulated (Figure 3; Figure 7), and expression of both homeologs of a possible ortholog of *ABR1*, a repressor of ABA signaling, is up-regulated (Figure S6). ABA interacts with *FUSCA3* which in turn regulates fatty acid biosynthesis and oil production [35,37]. The elevated expression in aflatoxin contaminated seeds combined with the very low expression in all other seeds free of contamination, even if infected with *Aspergillus*, points to this gene as a candidate susceptibility factor for pre-harvest contamination. This observation is strengthened by the parallel observation that ABA signaling, which this gene represses, is also down-regulated in contaminated seeds. 

The purpose of this study was to define the genetic state of the peanut seed that is permissive or repressive for aflatoxin production in the field. The expression data tell a story about the state of the peanut seed that permits aflatoxin contamination. The nature of this interaction in the field does not allow us to test whether the genetic state of the contaminated seeds was permissive of the aflatoxin contamination or a result of *Aspergillus* infection and subsequent contamination. From the sequence that we captured from the fungus itself, we found no evidence of aflatoxin cluster expression and therefore cannot say whether aflatoxin was being produced as we harvested (results not shown). We can, however, clearly show interacting pathways that are down-regulated in contaminated seeds. These pathways play important roles in seed development and maturity. Fatty acid biosynthesis, ABA signaling, sugar metabolism and transport, and cell wall modification all interact with each other during seed development. Regulators of these pathways, such as *WRINKLED1*, *FUSCA3*, or *ABI5* emerge as breeding targets to increase a cultivar’s ability to have low aflatoxin contamination under drought conditions. Perhaps the most intriguing target is *ABR1* as a possible susceptibility factor. These expression changes were not confirmed using additional methods, such as quantitative real-time PCR (qRT-PCR), and so future work must be done for confirmation. That being said, the results of this study suggest that the ability of the seed to develop under stress is a better breeding target for pre-harvest aflatoxin contamination than an enhanced defense response and that perhaps the fatty acid composition may play a significant role even in the field. 

## 4. Materials and Methods

### 4.1. Automated Rainout Shelter

An automated rainout shelter was used that is located at the Coastal Plain Experiment Station, Tifton, GA. The shelter has a fiberglass panel roof that can be opened and closed depending on the weather and contains 24 plots, each with two isolated 5-foot rows. The rooting zone (lower) is isolated from the pod zone (upper) by rubber sheets and sheet metal bent at a 90° angle. Each individual plot is equipped with four porous matrix or thermocouple psychrometer moisture sensors (Irrometer, Riverside, CA, USA) in the upper and lower soil zones coupled to a data logger that can open solenoid valves to minimally exceed the desired water activity for each plot. Each plot also contains temperature sensors (Irrometer, Riverside, CA, USA) that maintain the desired soil temperature by a thermostat-controlled harsh environment constant wattage heating cable in the pod zone. Each plot was filled with Tifton loamy sand, and inoculated with *Rhizobium* for nodulation.

The shelter was planted in a split plot design, with water treatment as the main plot factor and genotype as the sub plot factor. Six genotypes with varying maturities were used, two with medium–late maturity (Tifrunner and Florida-07) and four with medium maturity (Tifguard, C76-16, NC 3033, and A72). Based on preliminary data, these genotypes were chosen as having exhibited different levels of aflatoxin resistance from hyper-susceptible (A72) to moderately resistant (C76-16). Tifrunner, Tifguard, and Florida-07 are released cultivars [44,45,46] (Holbrook and Culbreath, 2007; Holbrook et al., 2008; Gorbet and Tillman, 2009). C76-16, A72, and NC 3033 are germplasm lines. C76-16 is a drought tolerant line that is a derivative of Tifton 8 [47]. NC 3033 is a breeding line shown to possess good resistance to soil borne fungal pathogens including *Cylindrocladium crotalariae* (CBR) [48]. A72 is a breeding line that has been determined to be aflatoxin hyper-susceptible. The two planting dates were May 16, 2015 (Tifrunner and Florida-07) and May 27, 2015 (Tifguard, C76-16, NC 3033, and A72) and each row was planted at three seeds per foot. At 45 days after planting (DAP), moisture sensors began maintaining the soil moisture in the root zone, just below field capacity. Soil temperature and moisture measurements were recorded every hour until harvest. At mid bloom, approximately 60 DAP, the plots were inoculated by manually spreading equal amounts on each plot of *A. flavus* NRRL3357 and *A. parasiticus* grown on cracked corn.

Treatments were started at 100 DAP of the last planting date and continued for 40 days. The three treatments were well-watered root and pod zone, well-watered root zone with heat- and drought-stressed pod zones, and heat- and drought-stressed root and pod zones. The soil temperature was maintained between 28–30 °C throughout the treatment. For RNA-seq analysis, individual seeds were harvested 140 DAP, quickly checked for maturity using the hull scrape method, and collected if demonstrating yellow-2 maturity stage. Selected seeds were shelled and each individual kernel was flash frozen in liquid nitrogen along with the pericarp. All seeds not selected were saved for plot level aflatoxin analysis.

### 4.2. Drought Tolerance Phenotyping

Three traits were measured as related to drought tolerance during the 40-day treatment period: canopy temperature depression, Normalized Difference Vegetation Index (NDVI), and visual stress rating. Canopy temperature was recorded by an infrared thermometer (Extech IR400, FLIR Commercial Systems, Nashua, NH, USA). NDVI estimates the photosynthetic output by measuring the proportion of visual light absorbed minus near infrared light reflected from the canopy. Canopy temperature depression is the temperature of the canopy related to the ambient temperature and is an indicator of tolerance under drought conditions. Additionally, moisture readings for each plot in the root and pod zones were taken every hour for the entire season. Four measurements along each row were taken at approximately 45° from the horizontal plane at approximately noon. Measurements were taken August 24, 28, September 9, 11, 16, 18, and 26 of 2015. Canopy temperature depression was calculated by using the readings from four ambient air temperature sensors (Irrometer, Riverside, CA, USA) located on either end of the shelter during the noon hour that readings were taken. NDVI measurements were taken at 12:30 p.m. only during sunny days using a CropScan Multispectral Radiometer (CropScan, Inc., Rochester, MN, USA). To cover the whole plot, each plot was scanned slowly for a duration 30 s and then, the average reading was taken. Measurements were taken on August 26, 29, September 9, 11, 16, and 18. Visual stress measurements were recorded on a 1–5 scale: 1 was healthy, 2 was slight bending down of branches, 3 was whole plant bending downward with leaves starting to turn brown, 4 showed brittle leaves and drying of the upper canopy, 5 was a nearly dead, extremely wilted plant. Visual measurements were taken at 1:00 p.m. on August 24, 26, 27, 28, 29, September 5, 9, 11, 16, 18, and 26, 2015 corresponding to 0, 2, 3, 4, 5, 12, 16, 18, 23, 25, and 32 days after treatment was initiated. Daily AUDPC was calculated for each trait to measure stress progress during treatment.

### 4.3. Aflatoxin Quantification

The plot level aflatoxin content of harvested, dried seeds was measured with a standard Vicam immunoaffinity column fluorometry method (Vicam Aflatest, Milford, MA, USA) on 10 g of shelled yellow-2 stage peanuts. Seeds along with 1 g dry NaCl (Fisher Scientific, Waltham, MA, USA) were homogenized in a small Kitchenaid blender, mixed with 40 mL of methanol (histological grade, Fisher Scientific, Waltham, MA, USA)/water (80:20 *v*/*v*), and shaken at room temperature for one hour. The extract was then filtered through Fisher (Waltham, MA, USA) medium porosity (P5) filter paper (particle retention 5–10 µm). Five mL of the filtrate was diluted with 20 mL HPLC water then re-filtered. A 1-mL filtrate was purified using a Vicam immunoaffinity column containing aflatoxin B1-specific monoclonal antibody. Aaflatoxin was eluted with 1 mL methanol and was measured with the fluorometer, excitation λ = 365 nm.

Seeds selected for RNA-seq analysis and positive for AflR DNA were subjected to aflatoxin analysis using a Waters Acquity Ultra Performance Liquid Chromatography (UPLC) instrument at the National Peanut Laboratory in Dawson, GA, USA. Briefly, aflatoxin was extracted using the Vicam immunoaffinity column method, described above, scaled down for 100 mg of tissue. Aflatoxin in 1 mL methanol was sent to Dawson for aflatoxin measurement. Separation and quantitation of aflatoxins was performed using the UPLC instrument equipped with a matching UPLC H-class Quaternary Solvent Manager, UPLC Sample Manager, UPLC Fluorescent Detector (FLR), and an Acquity UPLC BEH C18 2.1 mm × 50 mm, 1.7 µm column. The mobile phase was composed of a water/MeOH/CH3CN (64:23:13, *v*/*v*/*v*) mixture, and the flow rate was 0.30 mL·min^−1^. The column was maintained at 35 °C in the system column heater. Concentrations of aflatoxins were determined by reference to peak areas of corresponding commercial standards (calibration curve). The detection limit for aflatoxins G1 and B1 was 0.1 ng/g and 0.01 ng/g for aflatoxins G2 and B2.

### 4.4. Seed Processing for RNA Sequencing

A total of 238 individual seeds at yellow-2 stage were collected and frozen at −80 °C. Each seed was subjected to a combined RNA/DNA extraction method as described in Dang and Chen (2013). Before extraction, frozen samples were ground in liquid nitrogen and split into two 100 mg samples. DNA from each seed was tested for the presence of *Aspergillus* using PCR with primers for the aflatoxin cluster master regulator *AflR* (Forward: 5′-TCGTCCTTATCGTTCTCAAGG-3′ Reverse: 5′-ACTGTTGCTACAGCTGCCACT-3′) and an *Arachis hypogaea* Actin (Forward: 5′-CGAAGGCCAACAGAGAAAAG-3′ Reverse: 5′-CAATACCAGTTGTGCGACCA-3′) as control for DNA quality. Table S3 shows results from the seed screening. Figure S7 shows an overview of the seed screening process.

### 4.5. RNA Sequencing

Samples were pooled according to *Aspergillus* presence, aflatoxin content, or lack of both (Table S4). RNAs from at least two seeds were pooled for each sample for two biological replicates per genotype and state of infection/contamination. The quality of RNA was checked with an Agilent Bioanalyzer. Stranded RNA-seq libraries were constructed using a KAPA Stranded RNA-Seq Library Preparation kit (KR0934-v1.13; Kapa Biosystems, Wilmington, MA, USA) and the Illumina set B indexes (Illumina, San diego, CA, USA). Sequencing was done on an Illumina HiSeq2500 (Illumina, San Diego, CA, USA on three lanes with 12 samples pooled per lane. Sequencing statistics are shown in Table S5. Sequence quality was determined using FastQC (http://www.bioinformatics.babraham.ac.uk/projects/fastqc/, v. 0.11.4 2015). Reads were trimmed based on nucleotide usage bias on the 5′ end.

### 4.6. Expression Analysis

Each sample was mapped to a reference set of *A. hypogaea* transcripts (NCBI BioProject PRJNA291488) that have been annotated previously. This set of transcripts was assembled using a genome-guided pipeline that distinguishes between A and B-derived transcripts [49]. Expression was quantified using RSEM [50] which uses Bowtie as an aligner [51] and accounts for multiple mapping using a maximum likelihood estimation to calculate the estimated contribution of a read to multiple transcripts. Counts were used for a differential expression analysis using DESeq2 [52], using the “LRT” test which is a Likelihood Ratio Test where the null hypothesis is not different between the full and reduced linear models. Two models were used as follows: Full − Gene~Genotype + Treatment Reduced − Gene~Genotype to test the effect of treatment on gene expression while taking into account genotype. For tests of individual genotypes, the default procedure of DESeq2 was used. When using mixed genotypes, in the case of testing the samples with aflatoxin and *Aspergillus* present against the samples with *Aspergillus* present and no aflatoxin, the samples with identical states were treated as replicates.

### 4.7. Grouping of Differentially Expressed Genes and Enriched GO Analysis

Differentially expressed genes were grouped by expression profile using the Self-Organizing Maps (SOM) and the kohonen package in *R* (v 3.2.3; 2015) [53]. The expression profile for each gene was calculated by using FPKM values for expression and performing a *Z*-score normalization. The reference set of transcripts used were previously annotated using Trinotate [54] which included GO terms. A hypergeometric test for enrichment was carried out using the *R* function phyper and each *p*-value was adjusted for multiple testing using a Benjamini–Hochberg correction. Adjusted *p*-values were further filtered to be less than 0.001 to control for false positives due to smaller sample size. 

### 4.8. Non-Coding RNAs

A set of 6274 non-coding RNAs (ncRNAs) were included with the reference set of transcripts. Briefly, transcripts with no predicted open reading frame were subjected to further analysis. First, transcripts with an average FPKM lower than 2 were filtered out. Possible open reading frames (ORFs) were predicted again using the Coding Potential Calculator (CPC) [55] and transcripts with predicted ORFs longer than 80 amino acids were filtered out. Finally, coding potential was calculated again using a Coding Potential Assessment Tool (CPAT) [56] and 298 transcripts were estimated to have coding potential and eliminated. Alignment of ncRNAs in relation to associated genes was evaluated using BLAST and the annotated gene models of the diploid A and B genomes, *A. duranensis* and *A. ipaensis*, respectively (peanutbase.org; Ames, IA, USA).

### 4.9. Statistical Analysis

Differences between genotypes of drought tolerance traits were found using a Kruskal–Wallis test followed by a posthoc Dunn’s test with *p*-values adjusted with a Benjamini–Hochberg multiple testing correction using the PMCMR package in R [57]. Aflatoxin contamination in parts per billion (ppb) was natural log transformed to stabilize variance as in [8]. Linear modelling for natural log transformed aflatoxin was carried out using the lm (function in R. The best predictive model was used to predict aflatoxin values using the factors genotype, average root moisture, and ∆Ct with the predict function. 

### 4.10. SNP Identification

SNPs were identified using SWEEP (Clevenger and Ozias-Akins, 2015) with the following command, “perl SWEEP_Alpha.pl-b bamfiles.bam-g transcriptome.fa-o results.vcf-s 1-d 5-r 0—no_cleanup –ultimate.” The –ultimate filter checks each homozygous reference call for an alternative base and filters those out. The ‘-s’ parameter sets the genotypic likelihood filtering to medium stringency and the ‘-d’ parameter sets the per sample depth minimum to five reads covering a SNP. The rest are default parameters. 

## Figures and Tables

**Table 1:** Results of the differential expression analysis. Control indicates *Aspergillus* negative. Effect tested shows the effect that was tested for differential expression. DE genes = differentially expressed genes in each comparison.

Comparison	Effect Testing	Genotypes	DE Genes
*A. flavus* invasion			
*Aspergillus* present vs. Control	*A. flavus*	All	8
*Aspergillus* present vs. Control	*A. flavus*	C76-16	0
*Aspergillus* present vs. Control	*A. flavus*	NC 3033	0
*Aspergillus* present vs. Control	*A. flavus*	Tifguard	45
Aflatoxin production			
Aflatoxin present vs. Control	Aflatoxin/*A. flavus*	A72, Tifrunner, Florida-07	543
Aflatoxin present vs. not present	Aflatoxin	mixed	506
Aflatoxin present vs. Control	Aflatoxin/*A. flavus*	A72	28
Aflatoxin present vs. Control	Aflatoxin/*A. flavus*	Tifrunner	520
Aflatoxin present vs. Control	Aflatoxin/*A. flavus*	Florida07	1347

**Table 2:** Possible SNPs associated with expression changes in response to *Aspergillus*/aflatoxin. Fold change is the expression in FPKM in seeds not contaminated with *Aspergillus* or aflatoxin divided by seeds contaminated with *Aspergillus* or aflatoxin. Other genotypes reflect average fold change and standard deviation. All SNPs are located in a coding region from estimates using FGENESH, a program for predicting genes in genomic DNA sequences (http://www.softberry.com/berry.phtml?topic=index&group=programs&subgroup=gfind).

Fold Change (FC)	Other Genotype’s FC	Other Genotypes’ Base	Florida 07 Base	Annotation
832.23	69.46 ± 147.28	G	A	Bidirectional sugar transporter
399.89	4.85 ± 6.81	C	T	Beta-galactosidase 3
147.30	4.37 ± 6.99	C	T	Cannabidiolic acid synthase-like 1
103.33	1.84 ± 0.67	G	C	Kinesin-like calmodulin-binding protein
26.68	1.76 ± 0.83	A	T	(+)-neomenthol dehydrogenase
23.95	1.34 ± 0.70	C	A	Plasma membrane ATPase 4
23.41	1.59 ± 0.69	C	G	ATP synthase subunit a, chloroplastic
18.49	1.77 ± 1.61	G	A	Protein argonaute 5
18.48	1.85 ± 0.87	G	C	Receptor-like protein kinase HSL1
-	-	-	Tifrunner base	-
0.29	0.92 ± 1.01	A	T	Alpha-L-fucosidase 2
0.37	0.84 ± 0.42	T	C	Cytochrome P450 82C2

## Supplementary Materials

The following are available online at www.mdpi.com/2072-6651/8/11/317/s1, Figure S1: PCA analysis of drought-related traits, environmental conditions (moisture), and aflatoxin contamination, Figure S2: Prediction of Aflatoxin values using linear model, Aflatoxin ~ Genotype + Ct + Root Moisture, Figure S3: Expression in FPKM of transcripts associated with Potential eQTL, Figure S4: Expression in FPKM of fatty acid biosynthesis enzymes down-regulated in aflatoxin contaminated seeds, Figure S5: Expression in FPKM of fatty acid biosynthesis enzymes in seeds not contaminated with aflatoxin but infected with *Aspergillus*, Figure S6: Expression in *FPKM* of *ABR1*, Figure S7. Overview of seed screening process for selection for RNA sequencing, Table S1: Models Predicting Aflatoxin, Table S2: non-coding RNAs, Table S3: Seed Screening Results, Table S4: RNA Sequence Pooling, Table S5: RNAseq Read Stats.
