# Supplementary material for: RNA Sequencing of Contaminated Seeds Reveals the State of the Seed Permissive for Pre-Harvest Aflatoxin Contamination and Points to a Potential Susceptibility Factor
Source: Toxins (Basel). 2016 Nov 3;8(11):317. doi: 10.3390/toxins8110317 (PMC5127114; doi:10.3390/toxins8110317)
Supplement: Supplementary file 1 [file toxins-08-00317-s001.zip › toxins-152134 Supplementary for publish/toxins-152134-Figures S1-S7.docx]

Supplementary Materials: RNA Sequencing of Contaminated Seeds Reveals the State of the Seed Permissive for Pre-Harvest Aflatoxin Contamination and Points to a Potential Susceptibility Factor

Josh Clevenger, Kathleen Marasigan, Vasileios Liakos, Victor Sobolev, George Vellidis,
Corley Holbrook and Peggy Ozias-Akins


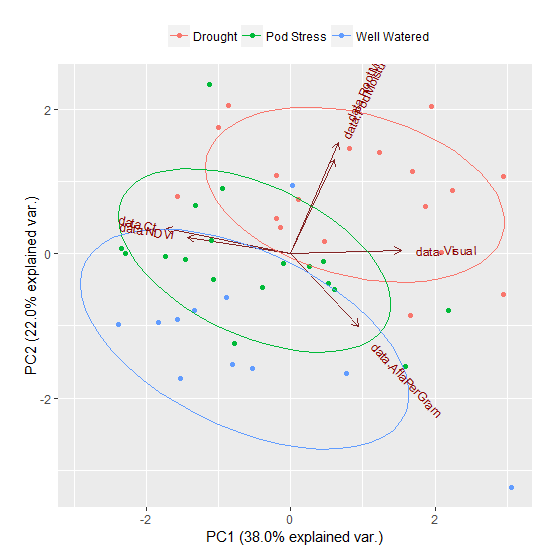


**Figure S1.** PCA analysis of drought-related traits, environmental conditions (moisture), and aflatoxin contamination. Points are rows indicated by treatment.


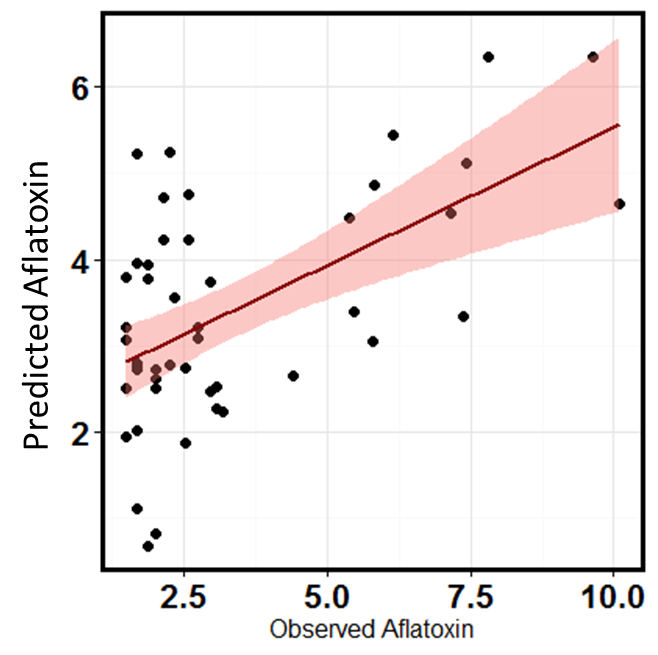


**Figure S2.** Prediction of Aflatoxin values using linear model, Aflatoxin~Genotype + Ct + Root Moisture. Regression line with *R^2^* = 0.32% and 95% confidence intervals. *Y* axis is predicted natural log transformed aflatoxin. *X* axis is observed aflatoxin.


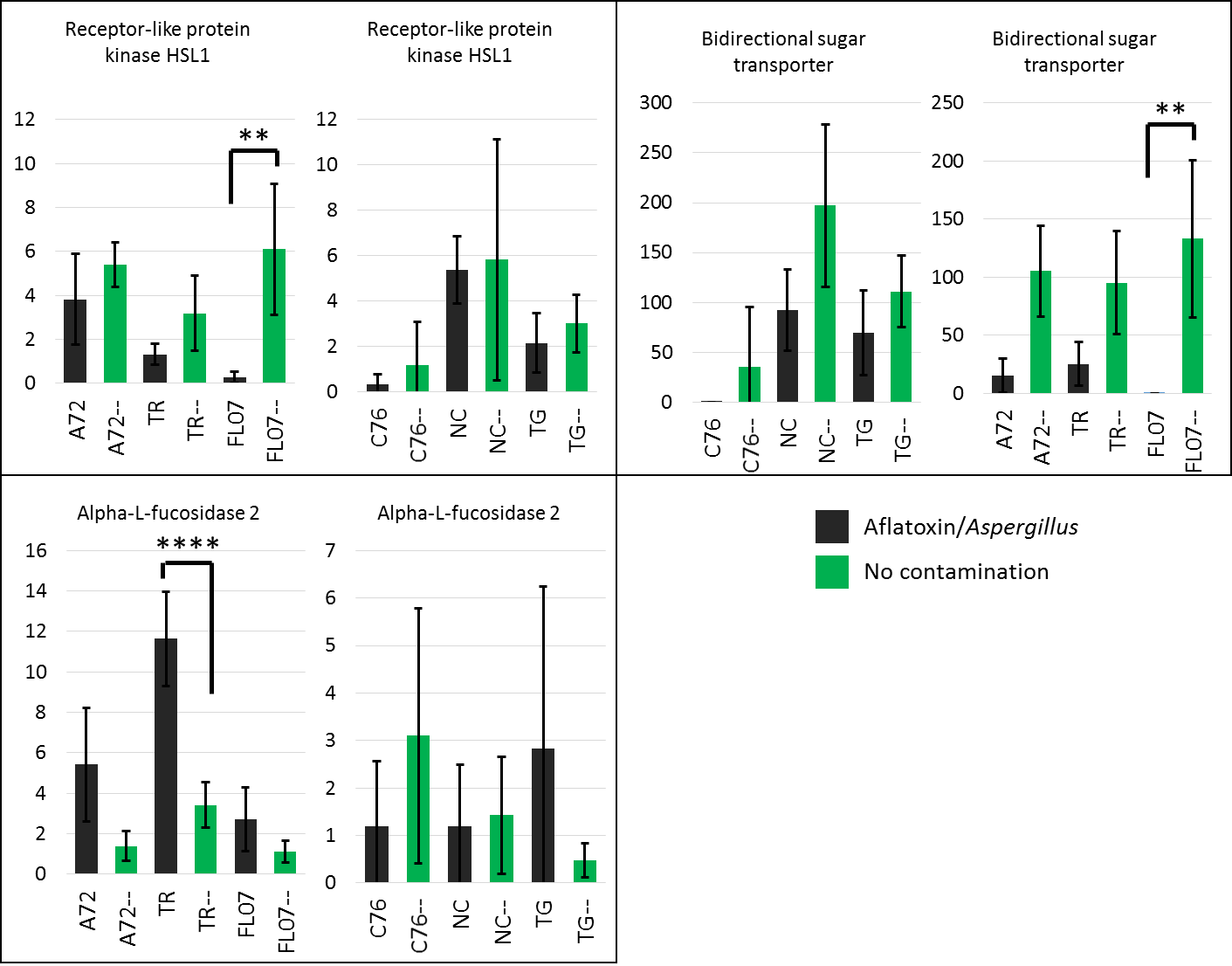


**Figure S3.** Expression in FPKM of transcripts associated with Potential eQTL. Graphs are average of biological replicates with error bars showing standard error. ** adjusted *p* < 0.05; **** adjusted *p* < 0.0001.


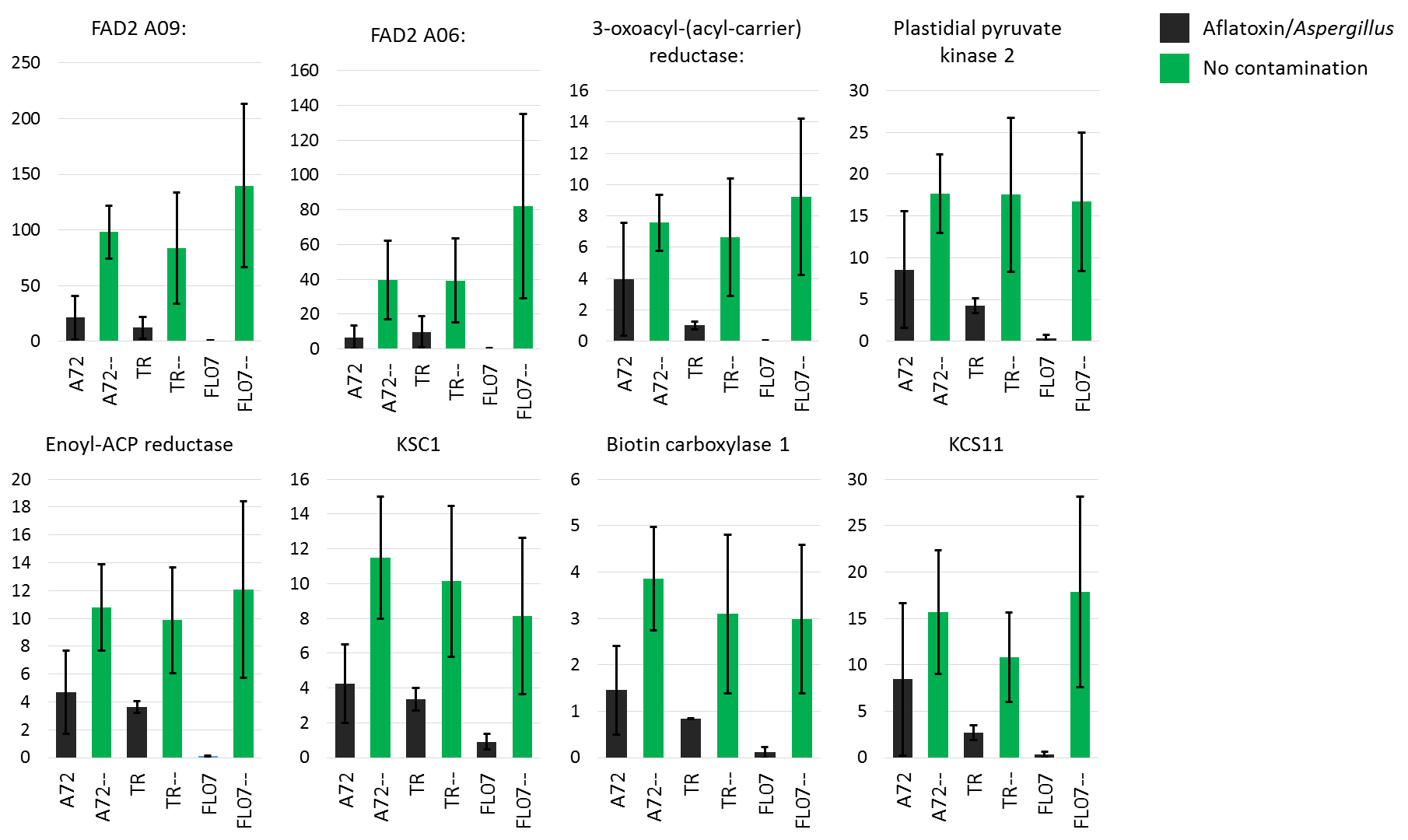


**Figure S4.** Expression in FPKM of fatty acid biosynthesis enzymes down-regulated in aflatoxin contaminated seeds.


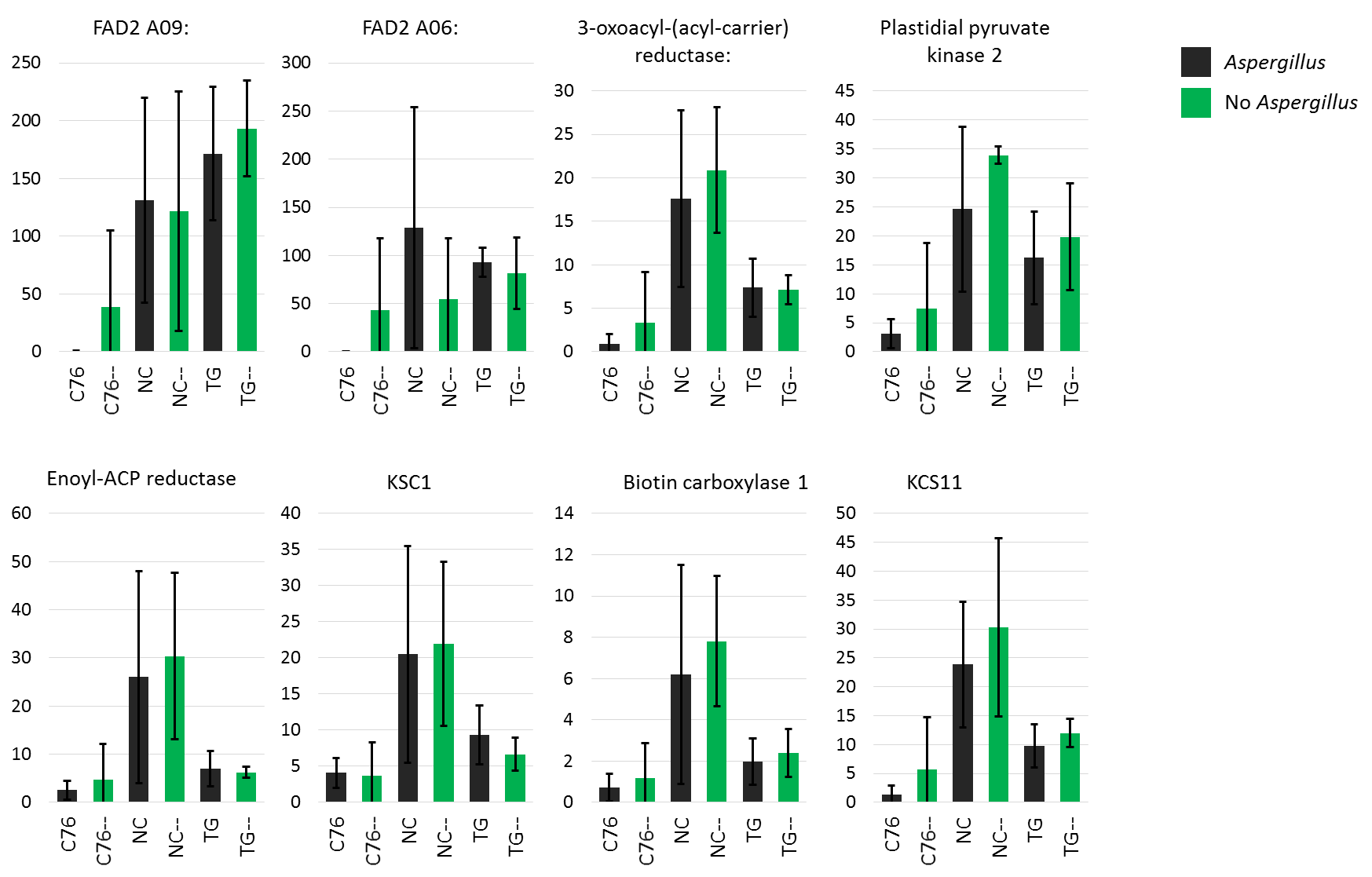


**Figure S5.** Expression in FPKM of fatty acid biosynthesis enzymes in seeds not contaminated with aflatoxin but infected with *Aspergillus*.


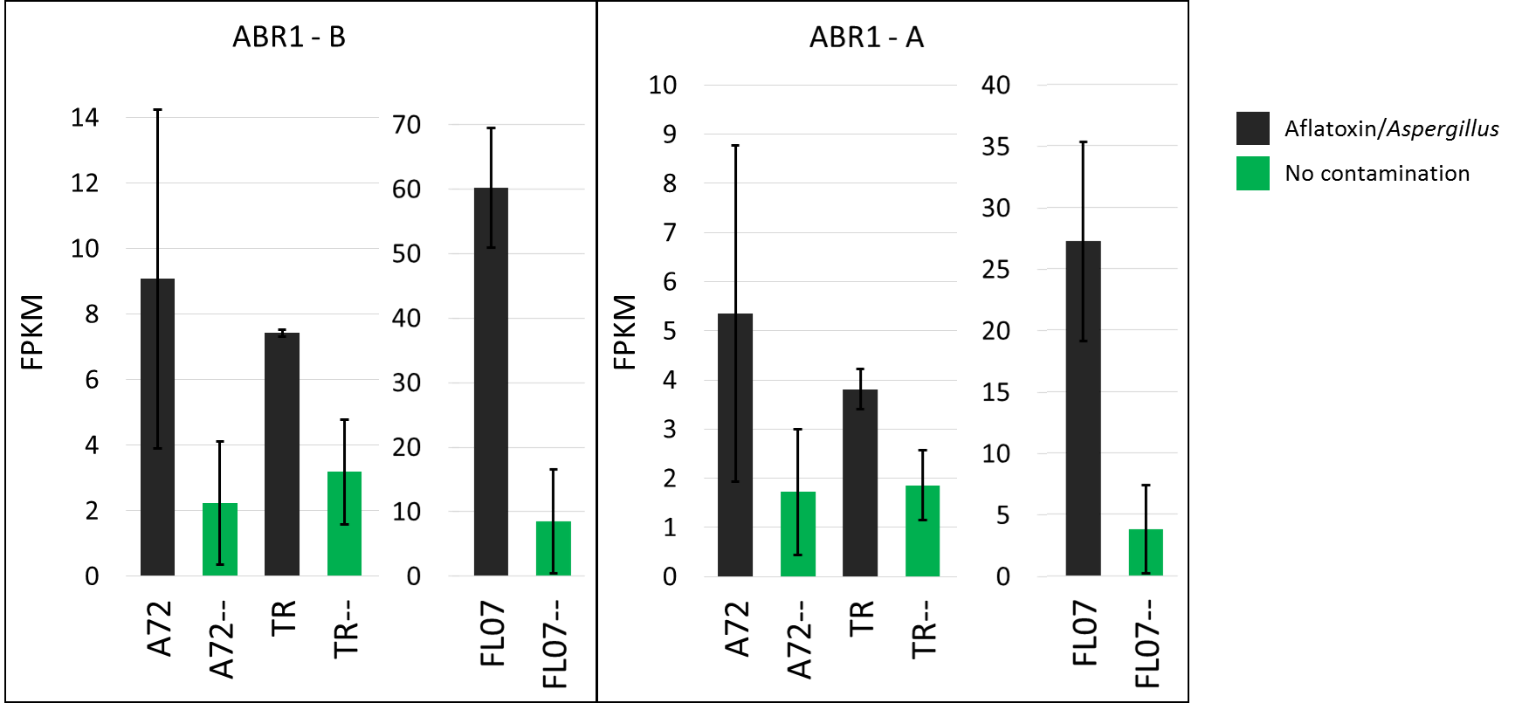


**Figure S6.** Expression in FPKM of *ABR1*. Graphs are average of biological replicates with error bars standard error.


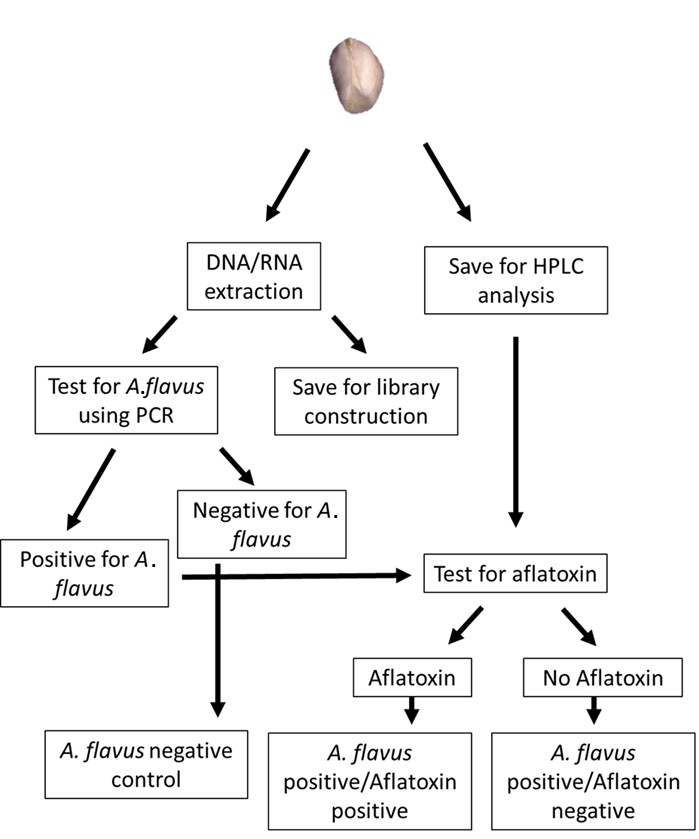


**Figure S7.** Overview of seed screening process for selection for RNA sequencing.
